# A Simulation Study of Debris Removal Process in Ultrasonic Vibration Assisted Electrical Discharge Machining (EDM) of Deep Holes †

**DOI:** 10.3390/mi9080378

**Published:** 2018-07-30

**Authors:** Yu Liu, Hao Chang, Wenchao Zhang, Fujian Ma, Zhihua Sha, Shengfang Zhang

**Affiliations:** School of Mechanical Engineering, Dalian Jiaotong University, Dalian 116028, China; liuyu_ly12@126.com (Y.L.); C2669112070@163.com (H.C.); traum525@gmail.com (W.Z.); mafujianyx@163.com (F.M.); shirlysha@hotmail.com (Z.S.)

**Keywords:** ultrasonic vibration assisted EDM, debris removal process, deep holes

## Abstract

When machining a small hole with high aspect ratio in EDM, it is hard for the flushing liquid entering the bottom gap and the debris could hardly be removed, which results in the accumulation of debris and affects the machining efficiency and machining accuracy. The assisted ultrasonic vibration can improve the removal of debris in the gap. Based on dynamics simulation software, Fluent, a three-dimensional (3D) model of debris movement in the gap flow field of EDM small hole machining assisted with side flushing and ultrasonic vibration is established in this paper. The laws of different amplitudes and frequencies and different aspect ratios on debris distribution and movement are quantitatively analyzed. The motion height of debris was observed under different conditions. The research results show that periodic ultrasonic vibration can promote the movement of debris, which is beneficial to the removal of debris in the machining gap. When compared to traditional small hole machining in EDM, the debris in the machining gap were greatly reduced, which ensures the stability of the machining process and improves the machining efficiency.

## 1. Introduction

Electrical discharge machining (EDM) as a non-traditional machining method uses the electro-thermal effect of pulse discharge between two electrodes to remove materials in dielectric fluid. As there is no mechanical contact between the tool and workpiece during the whole process, it does not produce mechanical cutting force and it is not restricted to mechanical properties of the strength of material and the hardness of material. Thus EDM has been successfully extended to the field of small hole [1]. However, a lot of debris are generated in the small hole EDM machining process. If the debris in machining gap are not removed in time, it will be accumulated and linked between the electrode and workpiece, which easily leads to an unstable machining process, such as the arc discharge and short circuit, affecting machining quality and material removal rate. Flushing is a common method for promoting debris removal in EDM operation, it is a process that makes dielectric fluid get into the machining gap, which will promote circulation of fluid, improve fluid mobility, and be beneficial to debris removal. But, the research indicates that when machining the hole with high aspect ratio, the large machining depth makes flushing difficult to enter the machining gap and to remove the debris. Through the relevant papers, it is found that the debris removal in the hole will be very difficult when the aspect ratio is more than 3 [2]. When the aspect ratio of hole is large, the problem of debris removal can be solved with the help of ultrasonic vibration.

Aimed at the studies of debris distribution in EDM of deep holes, Cetin et al. studied the effect of cutter lifting height on the debris movement with Star-CD. The simulation shows that it is very difficult to remove the debris from the interelectrode gap when the smaller cutter lifting height. When the cutter lifting height is large enough, the debris can be effectively removed from the side gap [3]. Wang et al. made researches on the debris and bubble movement with Fluent, proposed a three-dimensional model of flow field with liquid, gas, and solid phases for a machining gap in EDM. They summarized that the bubble that is generated at the bottom was the main factor to expel the debris. But, its ability became weak when the bubble reached the side gap [4,5]. Wang analyzed the effects of fluid pressures, drilling depth, and rotational speed of the tool on the debris concentration in high-speed small hole EDM drill. The simulation results show that the debris concentration increases with the drilling depth and increase of the fluid pressures can decrease the debris concentration. The rotational speed of tool, which ranges from 40 r/min to 120 r/min, has little effect on the debris concentration [6]. Ekmekci and Sayar studied the tip shape of tool under different machining conditions, it was observed that debris that was produced during machining accumulated at the tip, formed a hill and functioned as a tool electrode, especially when using fine machining condition [7]. Mastud et al. set up a simulation model of debris movement with ultrasonic vibration. The study found that the amplitude and frequency make the effect of debris movement and accelerate the debris movement [8]. Chang et al. studied the pressure field, velocity field, and the concentration of debris in machining gap during a period of ultrasonic vibration with Fluent. Simulation results show that ultrasonic vibration is beneficial to the removal of machining debris from inter-electrode gap [9]. Murray et al. centrifuged and imaged the debris set as negative polarity using SEM and TEM, made a research on the effect of debris on the electric field strength and predicted the trend of electric field strength increasing against debris concentration [10]. Pattabhiraman et al. developed a computational model to investigate the flushing of EDM debris from the interelectrode gap during the spray-EDM process. They pointed that the dielectric film thickness and velocity play a significant role in removing the debris from the machining region [11]. Tanjilul et al. studied an innovative simultaneous flushing and vacuum-assisted debris removal system, which facilitates better debris removal for deep-hole EDM drilling. The presented numerical model can be used to investigate the various factors that are influencing the removal of debris from the machining zone [12]. Besides, some researchers used tubular electrodes to gain the high aspect ratio deep holes. Ferraris et al. used the tools insulated on the sidewall by means of a coating to promote the process stability of micro EDM deep drilling by preventing secondary sparks. The achievable aspect ratio is increased with 30% and the tool wear is two times lower when using insulated electrodes [13]. D’Urso et al. dealt with the execution of through micro-holes using micro-EDM. The investigation focused on the influence of different electrodes’ materials and process parameters on both the process performance and the dimensional characteristics of the holes. The results showed that electrical resistivity, thermal conductivity, and melting point of both electrode and workpiece materials have a significant effect on the process and geometric performance [14,15]. Bellotti et al. investigated the micro-EDM process for drilling small through-holes on Ti-6Al-4V while using brass tubular electrodes. Taguchi L_32_ orthogonal array design and Gray relational analysis were proposed to select and optimize the process parameters that compromise between machining efficiency and surface finish [16]. Kliuev et al. analytically investigated the influence of drilling conditions to the pressure drop and dielectric flow during EDM drilling through computational fluid dynamics (CFD) simulations. The electrode diameter, gap, configuration of the flushing channel, electrode length, and drill depth were analyzed to estimate and describe the influence on the flow. The results showed that simple cylindrical flushing channel provides the best performance of the flow [17].

Given many available models, due to the debris movement in high aspect ratio hole EDM machining with ultrasonic vibration and side flushing is not yet fully understood. This paper develops a model to simulate the distribution and removal of debris in different machining conditions in ultrasonic assisted EDM with side flushing in fluid dynamical simulation software Fluent, and analyzes the influence of various parameters during the operation.

## 2. Establishing for the Model of Inter-Electrode Gap Field

**Figure 1:**
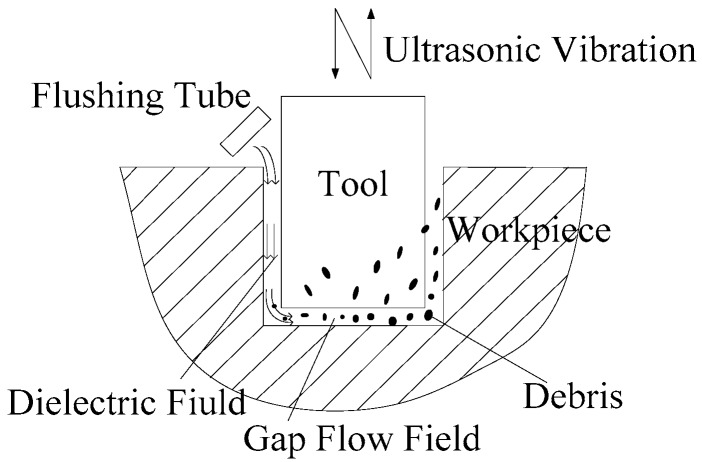
Process of debris motion in small hole machining of ultrasonic assisted electrical discharge machining (EDM).

The debris removal process in ultrasonic assisted EDM process for machining the small hole is shown in Figure 1. The flushing circulates the dielectric liquid in the bottom gap. Meanwhile, amplitude transformer drives the tool to do up and down reciprocating ultrasonic vibration. In this process, due to an enhancement of the cavitation effect, the electrode causes pressure difference, which increases the velocity gradient as well as the flow rate of working fluid in the bottom gap. Debris that are generated between two electrodes along with the working liquid is pushed to the region with lower pressure from the region with higher pressure. As the greater mobility of debris and the disturbance of tool vibration in the bottom gap, debris are removed from the bottom gap and side gap, finally removed out of the small hole.

### 2.1. Mathematical Model of Inter-Electrode Gap Flow Field

The drag force of particles (debris) are the most basic form of interaction between particles and fluid, which is the most significant parameter and must be taken into account when calculating. Our research assumes the conditions, as follows:

1. Particle (debris) is considered to be spherical.
2. The ambient temperature is considered as a constant and the simulation is not heat dissipates or exchanges.
3. The fluid field is considered to be infinite, inviscid and uncompressible. Fluid flows around the particles.

**Figure 2:**
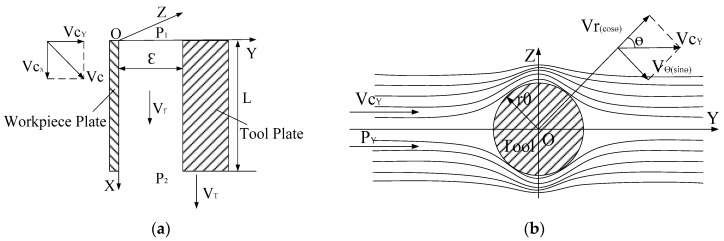
Schematics of inter-electrode gap. (**a**) The fluid speed along *x* axis; (**b**) The fluid speed along radial and circumferential.

In XOY coordinate, the side gap is regarded as two parallel plates. The right side of plate represents the wall of the tool and the opposite plate represents the inner surface of the hole. $v_{c}$ is the flushing and we will decompose it in two one-dimensional motions, one in the *x* direction ($v_{c_{x}}$), and one in the *y* direction ($v_{c_{y}}$). It is expressed as $v_{c_{x}}=v_{c_{y}}=\frac{v_{c}}{\sqrt{2}}$. $v_{T}$ and $v_{c_{x}}$ stand for tool speed and liquid speed along *x* axis, respectively, as shown in Figure 2a. In YOZ coordinate, the side gap is regarded as the model that ideal fluid flows around a cylindrical tool electrode and the distribution of velocity in YOZ coordinate plane is heterogeneous. The radius of cylindrical tool electrode is $r_{0}$. We will decompose $v_{c_{y}}$ in two one-dimensional motions, one in the radial direction of cylindrical tool electrode ($v_{r}(\cos\theta )$), and one in the circumference direction ($v_{\theta }(\sin\theta )$), as shown in Figure 2b.

#### 2.1.1. The Fluid Speed along Radial and Circumferential Direction

In polar coordinates, $v_{r}$ is the fluid speed along radial. $v_{\theta }$ is the circumferential speed. The relation between velocity distribution and velocity potential of the fluid around a cylindrical tool electrode are listed, as follows [18]:

(1) $$\begin{array}{l} v_{r}=\frac{\partial \;\varphi }{\partial r}=v_{c_{y}}(1-\frac{{r_{0}}^{2}}{r^{2}})\cdot \cos\theta \\ v_{\theta }=\frac{1}{r}\cdot \frac{\partial \;\varphi }{\partial r}=-v_{c_{y}}(1+\frac{{r_{0}}^{2}}{r^{2}})\cdot \sin\theta \end{array}\;$$

where *r* is the distance between spatial position and origin of coordinate. *θ* is the included angle between line *r* and positive *y*-axis. *φ* is the velocity potential in inter-electrode gap. Oseen summarizes a formula to get the drag force on the particles, which can be presented as [19]:

(2) $$F_{d}=6\pi \mu r_{p}\left(v_{f}-v_{p}\right)\sigma \;$$

Because the fluid speed along radial and circumferential speed is equated, respectively, with the liquid speed ($v_{f}$) in the YOZ coordinate, $F_{d_{r}}$ is the drag force along axial direction and $F_{d_{\theta }}$ is one along circumference direction. $r_{p}$ is the particle radius. We get the equation by combining the Equations (1) and (2):

(3) $$\begin{array}{l} F_{d_{r}}=6\pi \mu r_{p}\left(v_{c_{y}}(1-\frac{{r_{0}}^{2}}{r^{2}})\cdot \cos\theta -v_{p}\right)\sigma \\ F_{d_{\theta }}=6\pi \mu r_{p}\left(-v_{c_{y}}(1+\frac{{r_{0}}^{2}}{r^{2}})\cdot \sin\theta -v_{p}\right)\sigma \end{array}\;$$

#### 2.1.2. The Fluid Speed along *X* Axis

The liquid speed is considered for the effect of flushing ($v_{c_{x}}$) and tool movement. The tool speed ($v_{T}$) is expressed as the Equation (4).

(4) $$v_{T}=2\pi fA\cos\left(2\pi ft+\phi \right)\;$$

Naviers-Stokes equation can be simplified, as follows:

(5) $$-\frac{1}{\sigma }\frac{dp}{dx}+\lambda \frac{d^{2}v_{f}}{dy^{2}}=0\;$$

where *p* is the pressure, *x* and *y* are coordinates, *λ* is coefficient, and $v_{f}$ is the liquid speed along *x* axis. The pressure *p* along the *x* axis declines uniformly, which can be presented as:

(6) $$\frac{dp}{dx}=-\frac{p_{1}-p_{2}}{l}\;$$

where *p*_1_ − *p*_2_ is the pressure difference at both ends. When the tool moves down, $v_{T}$ points to the positive *X*-axis. As the tool moves up, $v_{T}$ points to the negative *X*-axis. We get the equation by combining the Equations (5) and (6):

(7) $$\frac{d^{2}v_{f}}{dy^{2}}=-\frac{p_{1}-p_{2}}{\sigma l\lambda }\;$$

By integrating the Equation (7), the fluid speed is obtained and it is listed, as follows:

(8) $$v_{f}=-\frac{p_{1}-p_{2}}{2\sigma l\lambda }y^{2}+C_{1}y+C_{2}\;$$

where *C*_1_ and *C*_2_ are constant coefficients.

The right side of plate (tool) has a speed of the ultrasonic vibration tool and it also drives the movement of the surrounding fluid. The left is still, finally, the boundary conditions are listed, as follows:

(9) $$\left\{ \begin{array}{l} y=\varepsilon ,v_{f}=v_{T}+v_{c_{x}}=2\pi fA\cos\left(2\pi ft+\phi \right)+v_{c_{x}}\\ y=0,v_{f}=0 \end{array}\right.\;$$

where *ε* is the *y*-axis position. *f* is the frequency of ultrasonic vibration, *A* is amplitude, and *t* is processing time. *C*_1_ and *C*_2_ can be solved by combining Equations (8) and (9). Substitute (2) for (8), therefore, the new formula can be shown and the drag force can be obtained by Equation (10):

(10) $$F_{d_{x}}=6\pi \mu r_{p}\left[-\frac{p_{1}-p_{2}}{2\sigma l\lambda }y^{2}+(\frac{v_{T}+v_{c_{x}}}{\varepsilon }-\frac{p_{1}-p_{2}}{2\sigma l\lambda }\cdot \varepsilon )y-v_{p}\right]\sigma \;$$

### 2.2. Modeling for the Inter-Electrode Gap Flow Field

**Figure 3:**
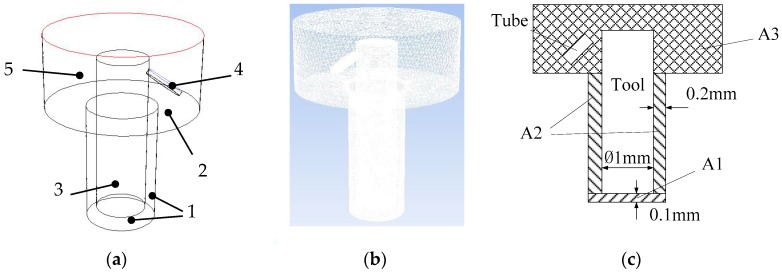
Model of the gap flow field in EDM. (**a**) Three-dimensional (3D) model; (**b**) 3D meshed model; and (**c**) Schematics of regions.

The gap flow field of small hole machining process is considered as a three-dimensional (3D) cylinder model that is established and meshed in the Gambit. The electrode and flushing are both cylinders. The machining depth is 3 mm. The tool electrode diameter is 1 mm. When considering the mobility of working fluid, the region above the electrode is appropriately much larger. Figure 3 is the model of the gap flow field in ultrasonic assisted EDM. In Figure 3, 1 is the wall of small hole, 2 is the surface of workpiece, 3 is tool electrode, 4 is flushing tube, and 5 is the flow region of workpiece surface. The flushing tube and working fluid need to exchange data, so an interface boundary is applied, pipe orifice is set to velocity-inlet, upper boundary is set to pressure outlet, and rest boundaries are set to walls as default. as shown in Figure 3a. The debris are generated near the electrode and workpiece. In order to improve the accuracy and efficiency of calculation, the grid near the bottom of electrode is finely meshed and rest regions are roughly meshed, as shown in Figure 3b. According to the previous research, the width of side gap is set to 0.2 mm and height of the bottom gap is set to 0.1 mm. Hence, the cylindrical region (A1) is the bottom gap, circular region (A2) is the side gap, and upper cylindrical region (A3) is the exterior flowing region, as shown in Figure 3c.

This paper uses Fluent to simulate the real machining process. The dynamic mesh technique provided by Fluent is used to solve the problem, so that the shape of flow field is constantly changing in response to the ultrasonic vibration of the tool electrode. In order to maintain a fine mesh quality, the smoothing and remeshing method are applied. The secondary development interface User Defined Functions (UDF) module is also used to simulate the process [20]. In order to simulate the generation of debris in different positions, functions are programmed to generate random numbers. In terms of the fact that the random function on the computer is a fake random, a function sleep for delaying is called every time when generating a random number. Then, *ranPosition* function is called to generate the random positions each discharge. The debris particle materials are copper and Ti alloy, respectively. Tool is red copper. Workpiece material is TC4. As the bottom of the electrode is applied with velocity-inlet, laminar-model is used to establish and simulate the flow field.

## 3. Simulation Results and Analysis

The simulation simulates the process of different amplitudes and frequencies within 0.04 s and contrastive analysis the distribution of the debris at different times. The simulation parameters in Fluent are set as Table 1. The simulation results are obtained.

### 3.1. Impact of Side Flushing on the Debris Removal

**Figure 4:**
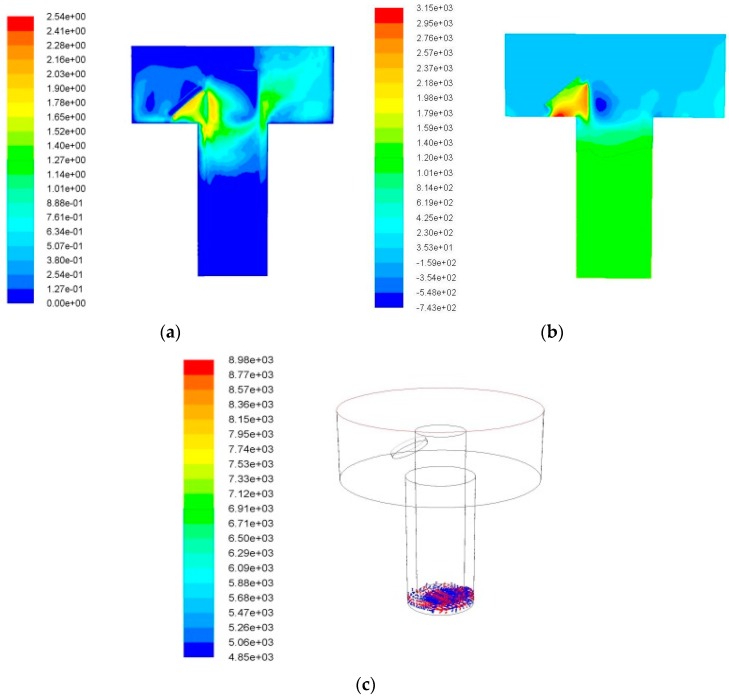
Machining without ultrasonic vibration. (**a**) Velocity field; (**b**) Pressure field; and (**c**) Debris distribution.

Figure 4 are the simulation results without ultrasonic vibration at 0.04 s. Figure 4a shows the velocity field, Figure 4b shows the pressure field, and Figure 4c shows the debris distribution.

It is shown in Figure 4a,b that, due to the side flushing action, a gap flow field is generated in which the fluids from the left gap near the flush inlet flow to the lower gap and then to the right side gap and at last flow out of the processing area. The highest fluid velocity of 2.54 m/s appears at the wall opposite to flushing direction. The fluid velocity of right side outlet is lower than that of left side inlet. The fluid velocity declines with the increase of hole depth. The highest pressure 3150 Pa appears at the inlet and the negative pressure zone also appears near the inlet, as shown in Figure 4b. However, due to the deep depth of the processing hole, the high speed working fluids formed by the flushing fluid cannot reach to the bottom effectively, and it can be found that the velocity of working fluids in the bottom gap is approximately 0 m/s. Therefore, it could be said that the side flushing has little effect on the debris removal in the case of deep hole machining. It is shown in Figure 4c that, after machining for a while, the debris are gathered together at the lower right corner and can not be removed from machining gap under the condition of side flushing without ultrasonic vibration. That is because the hole depth of machining is large, as the depth increases, the machining gap becomes a long narrow strip and the flowing resistance increases. It is hard for the flushing liquid to arrive at the bottom gap against resistance. This plays a weak role for debris movement of bottom gap, which results in the accumulation of debris and may cause the phenomenon of the arc discharge or short circuit. 

**Figure 5:**
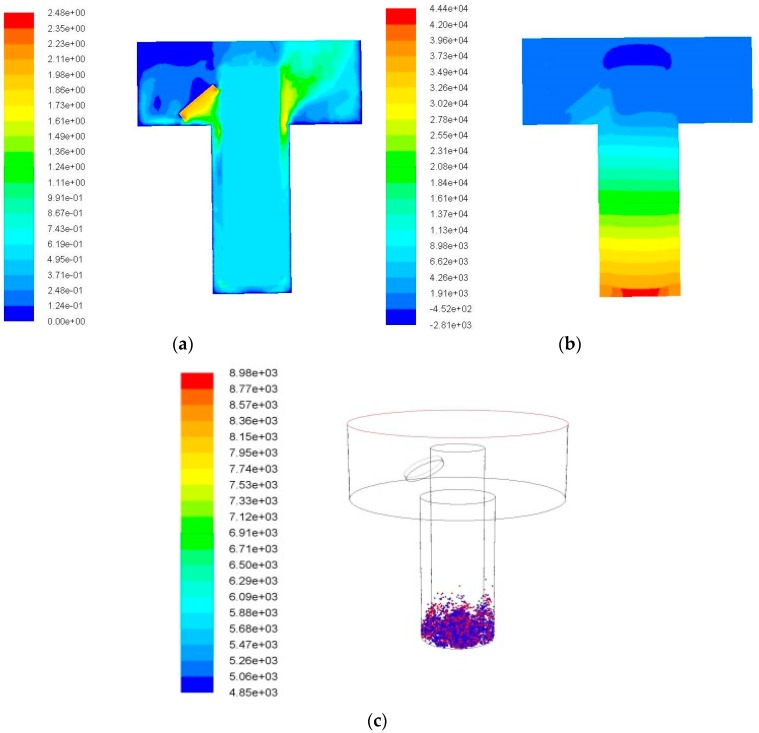
Machining with ultrasonic vibration. (**a**) Velocity field; (**b**) Pressure field; and (**c**) Debris distribution.

Figure 5 is the velocity field and pressure field distribution of debris with ultrasonic vibration. It is shown in Figure 5, that, in the case of ultrasonic vibration, the flow velocity and pressure of the working fluids in the bottom gap increases, which promotes the removal of the debris. Due to the effect of ultrasonic vibration, the flow velocity of the working fluid at the bottom of the deep hole reaches to about 0.5 m/s, and the highest pressure 44,400 Pa appears at the bottom of the hole, thus confirming the squeezing effect of ultrasonic vibration on the working liquid at the bottom. The debris are elevated to a certain height by the disturbance effect of ultrasonic vibration.

### 3.2. Impact of Vibration Amplitude on the Debris Removal

Ultrasonic vibration of the electrode is considered under the condition of side flushing. The motion states of the debris in the gap flow field under different amplitudes are simulated. The simulation results show that in the machining process, the distributions of debris in the gap flow field increase with time. The ultrasonic vibration frequency was 20 kHz, and the amplitude of the amplitude was 5 μm, 10 μm, and 20 μm.

**Figure 6:**
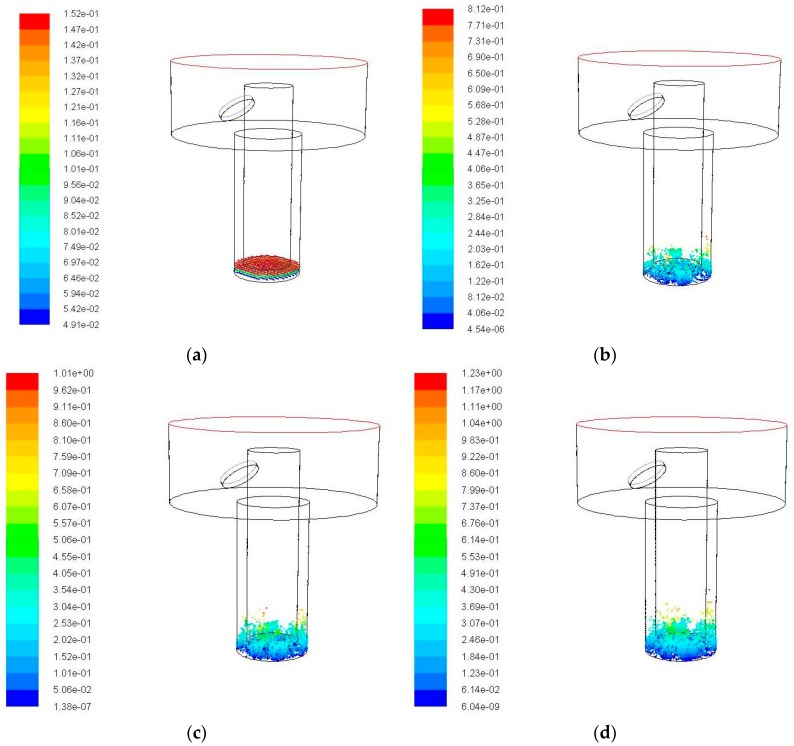

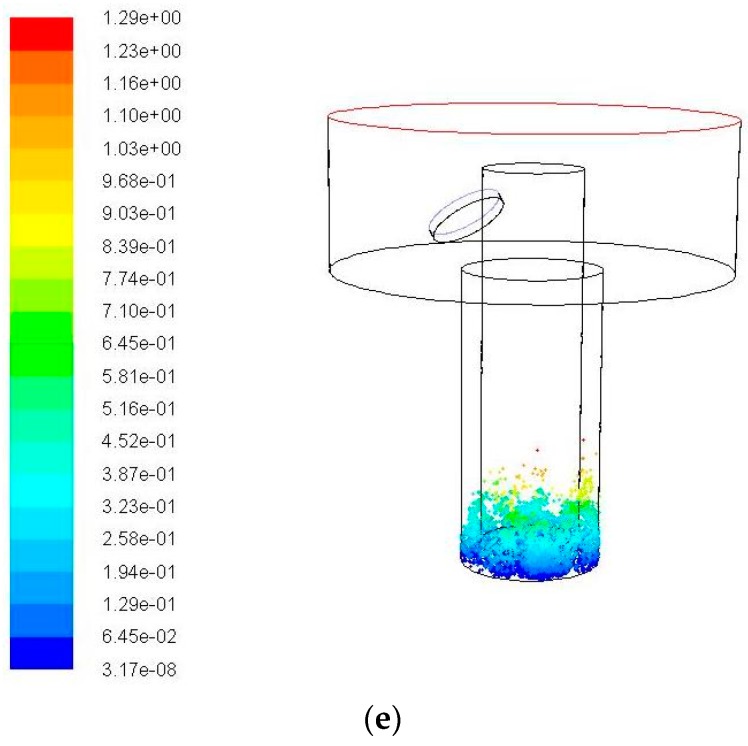
The debris movement with time when ultrasonic frequency is 20 kHz, amplitude is 5 μm. (**a**) Initial debris distribution; (**b**) Debris distribution at 0.01 s; (**c**) Debris distribution at 0.02 s; (**d**) Debris distribution at 0.03 s; and (**e**) Debris distribution at 0.04 s.

When the amplitude is set to 5 μm, the changes of the simulation result with time are shown in Figure 6. When compared with the simulation results without ultrasonic vibration of the electrode in Figure 5, the debris movements with ultrasonic vibration are more active. It can be seen from Figure 6 that a portion of the debris originally accumulated at the lower right corner is moved into the side gaps. In addition, the number of debris and the movement height increases with the time. The accumulation of debris is obviously reduced. This is because the ultrasonic vibration assisted electrode does the up-and-down piston motion. When the electrode moves up, a negative pressure region is generated in the bottom gap of electrode and the pressure gradient of entire gap flow field becomes large. The external working fluid flows into the bottom clearance, impacting and disturbing the debris to do the acute movements. When the electrode moves down, the working fluid in the machining gap is squeezed by the lower surface of electrode and moved from the bottom gap to side gap, and even out of the processing area. This process can make debris move towards the outlet of the hole.

As can be seen from Figure 6e, in 0.04 s, a large number of debris are brought to a certain height by the disturbance of ultrasonic vibration. However, it fails to reach the effective action area of the flushing liquid due to the limited height enhanced, the amount of debris was removed out of the processing hole or moved into the exterior flowing region is very small and can not be observed obviously. The maximum motion height of debris is 1.29 mm.

**Figure 7:**
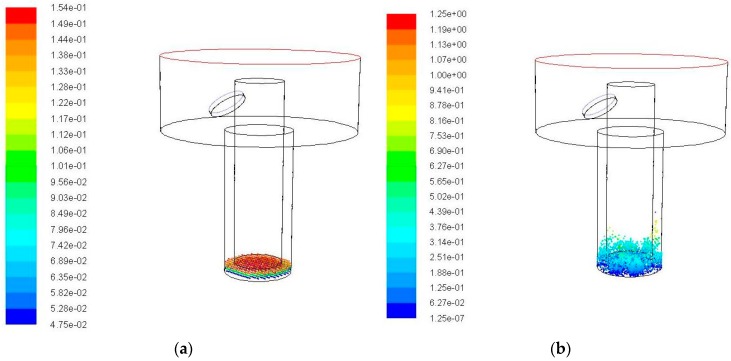

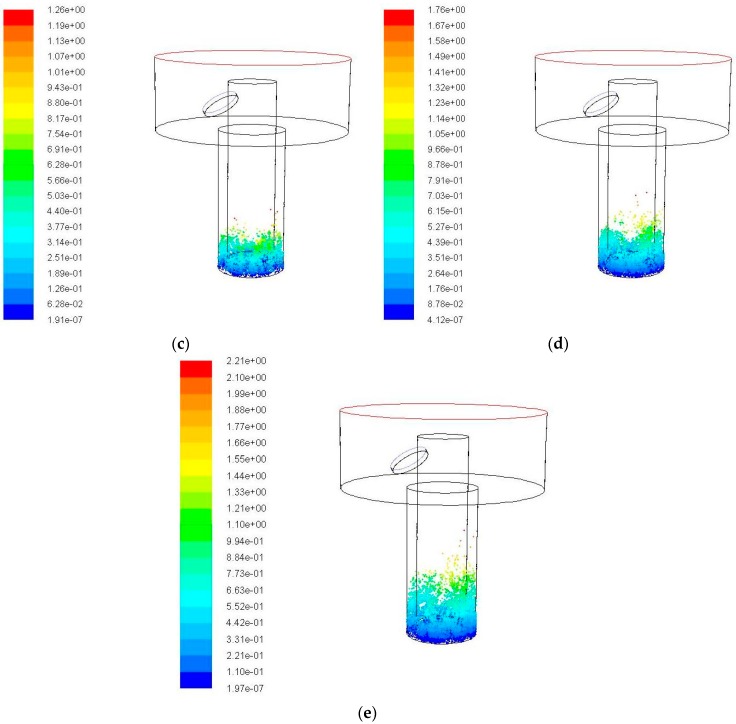
The debris movement with time when ultrasonic frequency is 20 kHz, amplitude is 10 μm. (**a**) Initial debris distribution; (**b**) Debris distribution at 0.01 s; (**c**) Debris distribution at 0.02 s; (**d**) Debris distribution at 0.03 s; and (**e**) Debris distribution at 0.04 s.

When the amplitude is increased to 10 μm, the simulation results of the debris movement with time are shown in Figure 7. When compared with Figure 6, when the amplitude is increased to 10 μm, it can be seen by comparing the position of debris at each moment in Figure 7 that the height of debris movement is increased with time, for example, at 0.04 s, the debris movement height is significantly higher than that in the case of 5 μm amplitude. The maximum motion height of debris is 2.21 mm. The number of debris in the side gap increases obviously, and it relatively decreases in the bottom gap. But, it is still not obvious to see the debris that is removed out of the processing hole.

**Figure 8:**
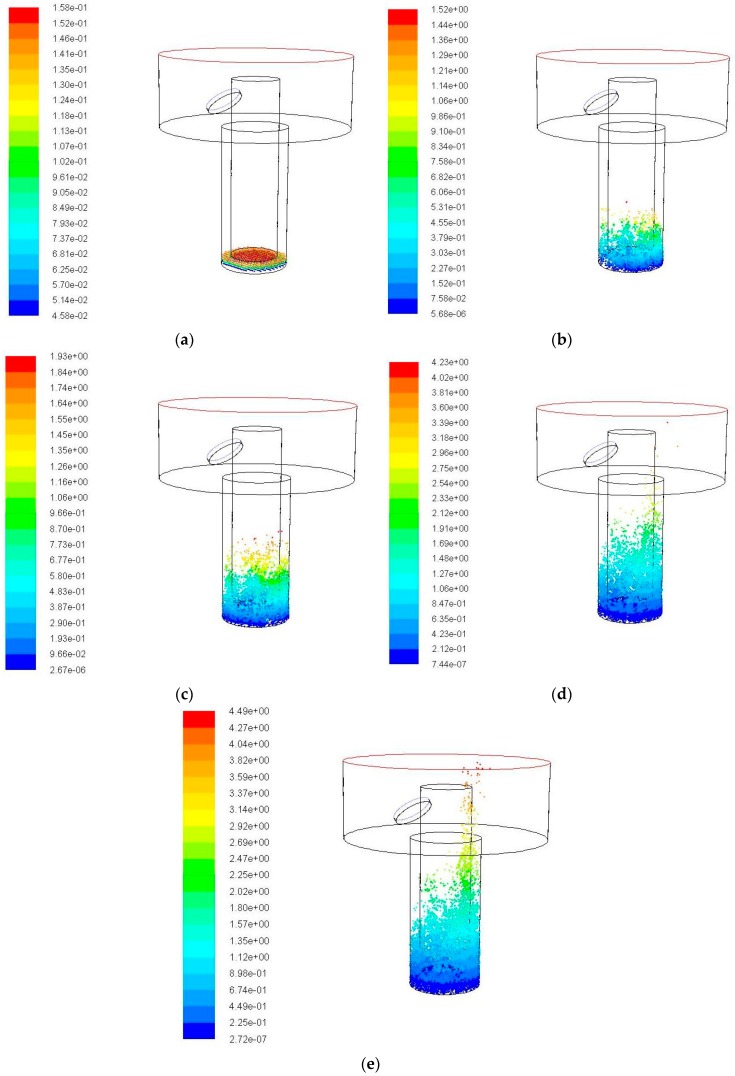
The debris movement with time when ultrasonic frequency is 20 kHz, amplitude is 20 μm. (**a**) Initial debris distribution; (**b**) Debris distribution at 0.01 s; (**c**) Debris distribution at 0.02 s; (**d**) Debris distribution at 0.03 s; and (**e**) Debris distribution at 0.04 s.

When the amplitude increases to 20 μm, the simulation results are shown in Figure 8. It can be clearly seen from Figure 8 that the number of debris in the side gap at each moment is significantly increased, and the number of remained debris in the bottom gap is significantly reduced, debris rise higher with time increase. The maximum motion height of debris is 4.49 mm. It can be found in the middle and later stage of the simulation that the debris are taken to the effective action area and it accumulates in the right gap under the influence of the flushing action and moves to the outlet of the hole. At 0.04 s, it is obvious that a large number of debris are removed out to the exterior flowing region.

Through comparing and analyzing three groups of simulation results, it can be found that ultrasonic vibration enlarges the effect of working fluid disturbance in the bottom gap. The larger the amplitude is, the more obviously the liquidity increases, and the higher the debris from the bottom can reach. When the debris are carried by ultrasonic vibration to the effective action area of the flushing fluids, the side flushing action can remove the debris from the discharge machining area. Increasing the amplitude of ultrasonic vibration makes the disturbance effect of the tool electrode on the debris increase in the bottom gap. It is beneficial to improve the carrying capacity of the working fluid to the debris, promoting the debris in the bottom gap removal out of the machining hole.

### 3.3. Impact of Vibration Frequency on the Debris Removal

When considering the effect of different frequency on the movement law of debris at the same amplitude, the amplitude is selected as 5 μm, and the frequency is 30 kHz and 40 kHz for simulation analysis. The simulation results are analyzed compared with that in the condition of 5 μm amplitude and 20 kHz frequency.

**Figure 9:**
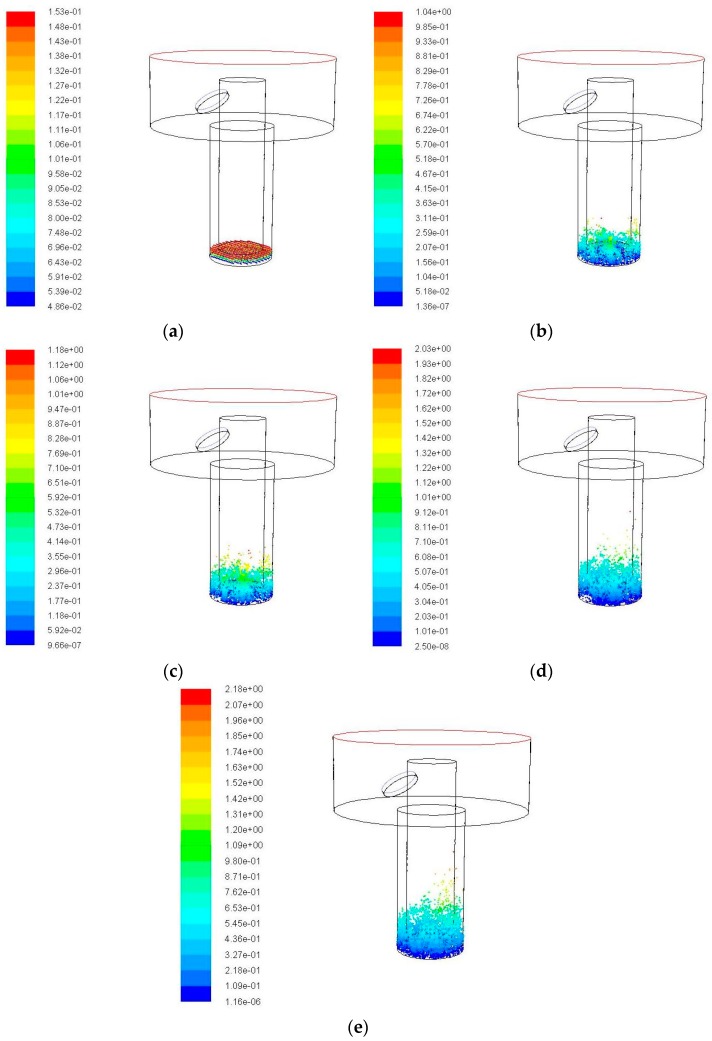
The debris movement with time when ultrasonic frequency is 30 kHz, amplitude is 5 μm. (**a**) Initial debris distribution; (**b**) Debris distribution at 0.01 s; (**c**) Debris distribution at 0.02 s; (**d**) Debris distribution at 0.03 s; and (**e**) Debris distribution at 0.04 s.

**Figure 10:**
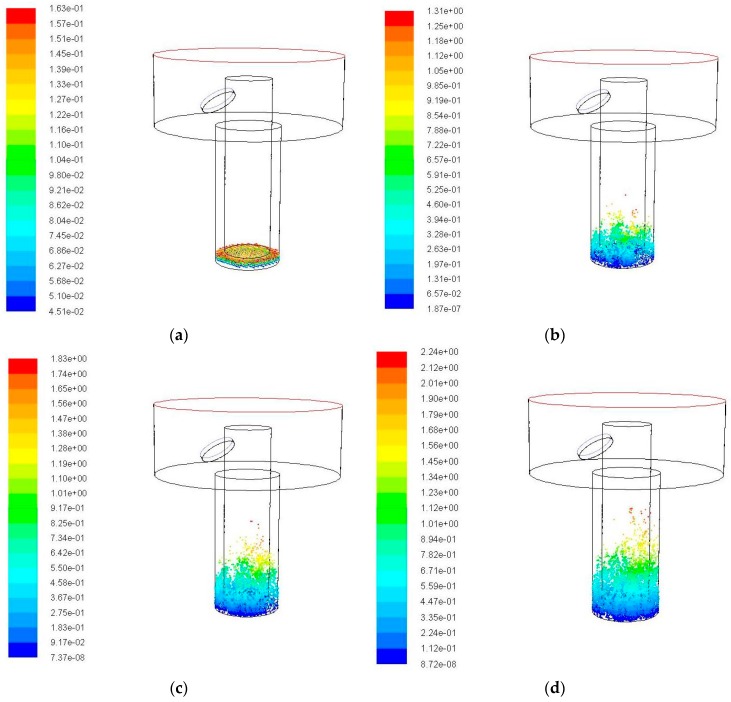

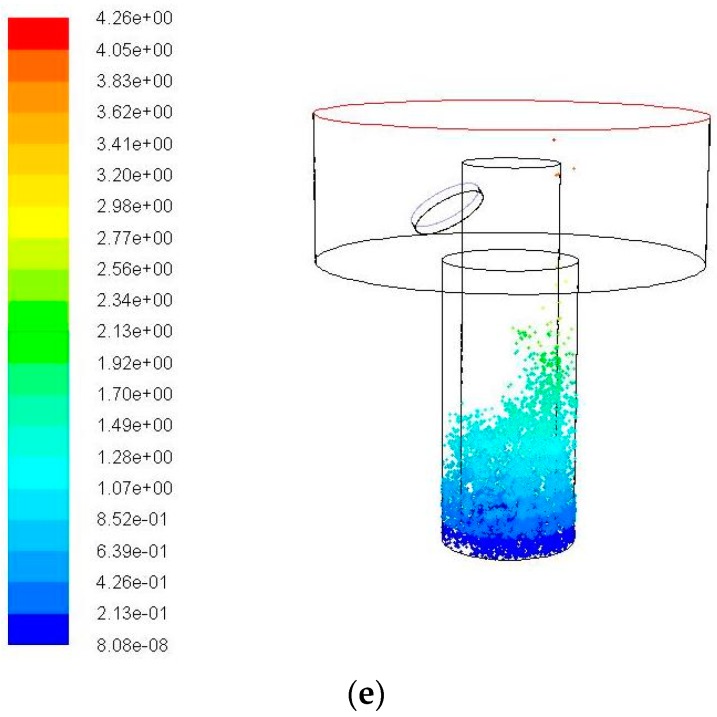
The debris movement with time when ultrasonic frequency is 40 kHz, amplitude is 5 μm. (**a**) Initial debris distribution; (**b**) Debris distribution at 0.01 s; (**c**) Debris distribution at 0.02 s; (**d**) Debris distribution at 0.03 s; and (**e**) Debris distribution at 0.04 s.

When the frequency is 30 kHz, the simulation results are shown in Figure 9. When compared with the simulation results in Figure 6, when the frequency increases to 30 kHz, the debris amount of the side gap at each moment is increased, but the increase is not obvious, as shown in Figure 10. The maximum motion height of debris is 2.18 mm. The debris are carried to a certain height, but the amount of debris removed into the exterior flowing region is very small.

When the frequency is 40 kHz, the simulation results are shown in Figure 10. As can be seen from Figure 10, when the vibration frequency increases to 40 kHz, in the discharge area, there is a large increase in the number of debris at each moment in the side gap, and the debris in the bottom gap decrease. At 0.04 s, it is obvious that a large number of debris are removed out of the machining hole. The maximum motion height of debris is 4.46 mm.

It can be obtained by analyzing that the debris movement becomes acuter and more disordered when the frequency increases. A large number of debris move from the bottom gap to the side gap. The higher the frequency is, the more disordered the debris are, and the higher the debris from the bottom can reach. As the frequency is higher, the number of longitudinal movements of the electrode in a unit time is increased and the disturbance of working fluid is acuter in the vertical direction, the debris removed out of the bottom gap are also more, following the working fluid. It is beneficial to the removal of debris, and the accumulation of debris is reduced.

### 3.4. Debris Removal under Different Hole Depths

**Figure 11:**
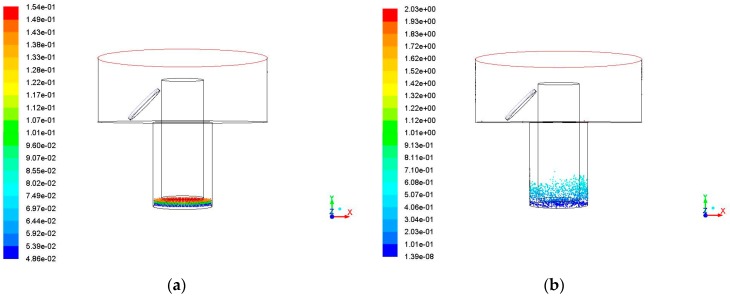

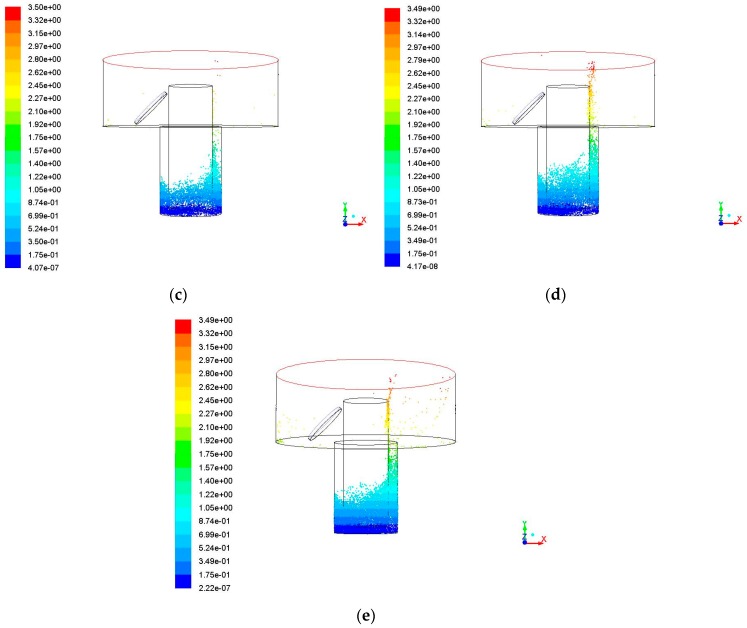
The debris movement with time when the hole depth is 2 mm, amplitude is 5 μm, frequency is 20 kHz. (**a**) Initial debris distribution; (**b**) Debris distribution at 0.01 s; (**c**) Debris distribution at 0.02 s; (**d**) Debris distribution at 0.03 s; and (**e**) Debris distribution at 0.04 s.

The simulation was carried out for different hole depth (2 mm and 4 mm) to verify the effect of flushing liquid and ultrasonic vibration on the removal of debris. The amplitude and the frequency were kept constant, 5 μm and 20 kHz respectively, as the already condition tested of 3 mm hole depth. Figure 11 shows the simulation results of the debris at different positions with time when the hole depth is 2 mm.

**Figure 12:**
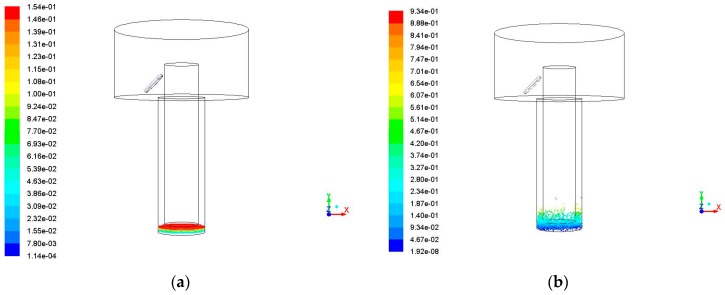

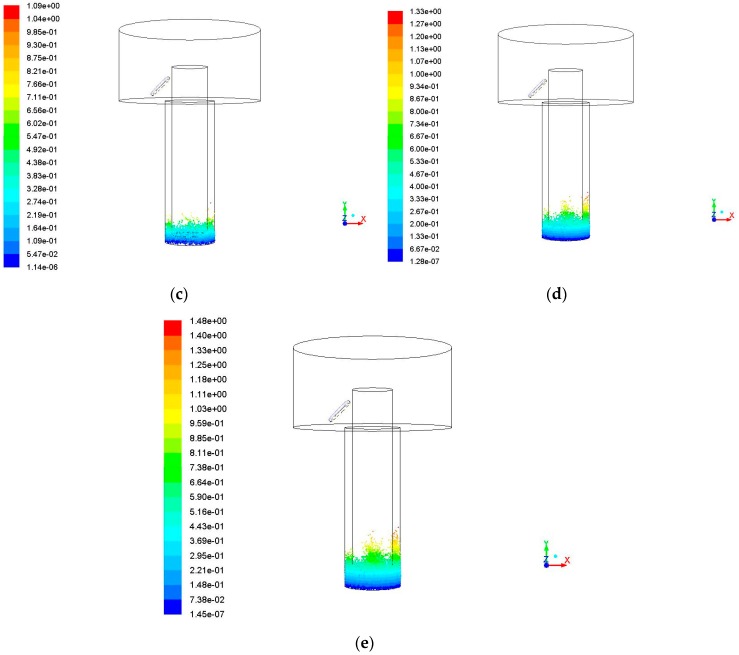
The debris movement with time when the hole depth is 4 mm, amplitude is 5 μm, frequency is 20 kHz. (**a**) Initial debris distribution; (**b**) Debris distribution at 0.01 s; (**c**) Debris distribution at 0.02 s; (**d**) Debris distribution at 0.03 s; and (**e**) Debris distribution at 0.04 s.

It can be seen from the simulation results that when the hole depth is 2 mm, when compared with the hole depth of 3 mm, the debris are easier to remove. At 0.02 s, a large number of debris have been removed away from the processing gap under the effects of ultrasonic vibration and flushing liquid. There is a trend of continuous delivery of the debris in the rest of the time. Figure 12 shows the simulation results of the debris at different positions with time when the hole depth is 4 mm.

When the hole depth increases to 4 mm, it can be seen from the simulation results that the removal efficiency of debris is decreased when compared to the depth of the hole is 3 mm at 0.04 s. This is because the effect of ultrasonic vibration on the debris is limited in a certain period of time, the debris rise to a certain height under the impact of ultrasonic vibration. However, the effective depth of the flushing liquid is limited due to the constant velocity of the flushing liquid, and the debris have not reached the effective area of the flushing liquid. As the processing time goes on, the height of debris in the lateral clearance is increasing under the impact of ultrasonic vibration. Once it reaches the effective areas, it will be easily removed.

## 4. Statistics and Analysis of Debris Distribution

After simulations, the position of each debris is exported to a text file from the Fluent, and a program is written to make statistics for the debris. The program reads the text file, including the debris information, such as position *x*, *y*, *z*, density and time steps. The range (*x*, *y*, *z*) of each region is input into the interface and every debris that satisfy the range will be selected and recorded to get the total number of the debris. In different amplitudes and frequencies, it is, respectively, counted that the quantity of debris in A1 region (bottom gap), A2 region (side gap), and A3 region (exterior flowing region) to analyze the results and to find out the relevant laws.

### 4.1. Impact of Vibration Amplitude on the Debris Distribution

**Figure 13:**
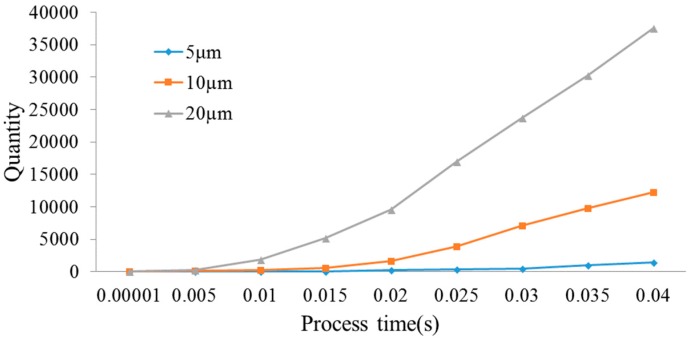
The quantity variation of debris.

When processing 3 mm deep hole and frequency with 20 kHz, Figure 13 presents the quantity of debris with the different amplitudes of 5 µm, 10 µm, and 20 µm, respectively, in A3 region. It is shown that the quantity of debris is gradually increased in A3 region as the process progresses. By observing the velocity of debris removal with the three kinds of amplitudes, it is found that all of the velocity increases first and then incline to a stable value. In addition, as the amplitude increases, the velocity of debris removed is remarkably faster. In unit time, the quantity of debris removed into the A3 region is also remarkably increased.

**Figure 14:**
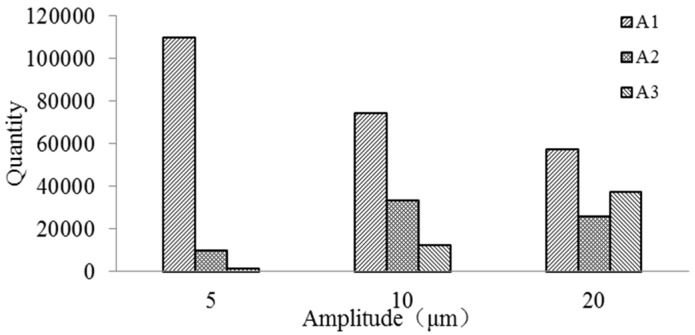
The quantity of debris distribution.

Figure 14 is the quantity of debris distribution with different amplitudes in A1, A2, and A3 regions at 0.04 s. It is shown that when the amplitude increases, the quantity of debris in A1 region steadily reduces, the quantity of debris in A2 region increases first and then inclines to stable value and the quantity of debris in the A3 region remarkably increases. According to the statistics, 121,356 debris are generated in the bottom gap. When the amplitude is 5 µm, the quantity of debris in A1 region is 109,776, the quantity of debris in A2 region is 10,115 that is relatively fewer and in A3 region is 1465, which is the fewest. The removal rate is 1.21% and the residual rate in the bottom gap is 90.46%. As the amplitude increases to 10 um, it is found that the quantity of debris reduces in the A1 region when comparing with the 5 µm and the quantity is 74,320. The quantity of debris in A2 region rises to 33,678 and the A3 region rises to 12,258. That removal rate is 10.1% and the residual rate is 61.24%. When amplitude increases to 20 µm, the quantity of debris in A1 region is fewer than that of 10 µm as 57,518. The A2 region declines to 25,949, the quantity of debris in A3 region obviously increases and the quantity of debris rises to 37,532. The removal rate is 30.9% and the residual rate in the bottom gap is 47.40%. Therefore, with the growth of amplitude, the debris in exterior flowing region significantly increases, the removal rate increases, debris that remain in the bottom gap obviously reduce, and the residual rate reduces. So, when the vibration amplitude considerably increases, it is beneficial to the debris removal from the machining gap as well as the improvement of machining efficiency of small hole machining in ultrasonic-assisted EDM.

### 4.2. Impact of Vibration Frequency on the Debris Distribution

**Figure 15:**
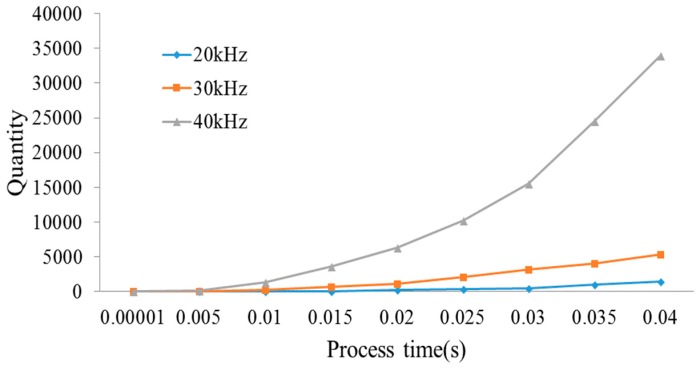
The quantity of debris.

**Figure 16:**
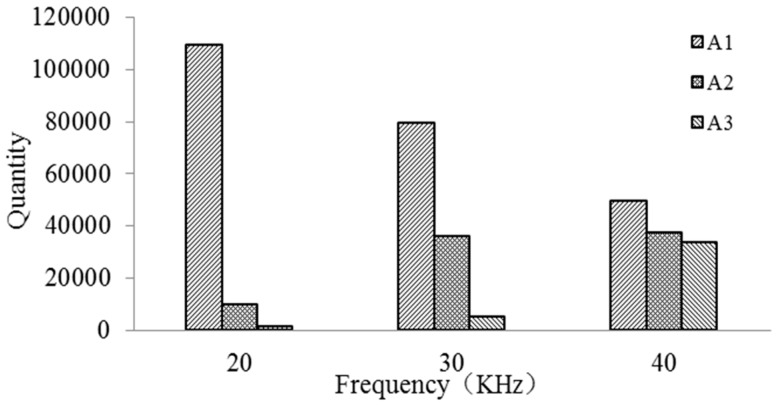
The quantity of debris distribution.

When the ultrasonic vibration amplitude is 5 µm, Figure 15, respectively, presents the quantity of debris with the frequencies of 20 kHz, 30 kHz and 40 kHz in A3 region. From Figure 16, it is observed that the quantity of debris in A3 region continuously grows from 0 to 0.04 s. By observing the velocities of debris removal of three frequencies, the velocities increase first and then inclines to a stable value. In addition, as the frequency increases, the velocity of debris removal is remarkably faster. When the frequency increases to 40 kHz, the velocity and quantity of debris removal quantity is significantly higher than the other two cases.

Figure 16 is the quantity of debris distribution with the frequencies of 20 kHz, 30 kHz, and 40 kHz in A1, A2, and A3 regions at the simulation time 0.04 s. The quantity of debris in the A1 region steadily reduces and the quantity in A2 region increases first and then inclines to stable value as a result of high frequency. In addition, the quantity in A3 region obviously increases. The laws of the quantity of debris in A1 region, A2 region, and A3 region are consistent with Figure 13. It is found that most of debris in the bottom gap move to the side gap and exterior flowing region as the vibration frequency increases. According to the statistical analysis, the removal rate is 1.21% when the frequency is 20 kHz and the removal rate increases to 4.37% when it is 30 kHz. When it is 40 kHz, the removal rate is 27.9%, which is much greater than the former cases. On the contrary, the higher frequency reduces residual rate of debris and they are, respectively, 90.45%, 65.77%, and 41.07%. So, it can be seen that higher frequency improves the removal rate and reduces the residual rate of debris. Most of debris are removed to the exterior flowing region avoiding the accumulation of debris. The results also indicate that the increasing vibration frequency has a significant effect on the removal of debris.

### 4.3. Effect of Hole Depth on the Distribution of Debris

**Figure 17:**
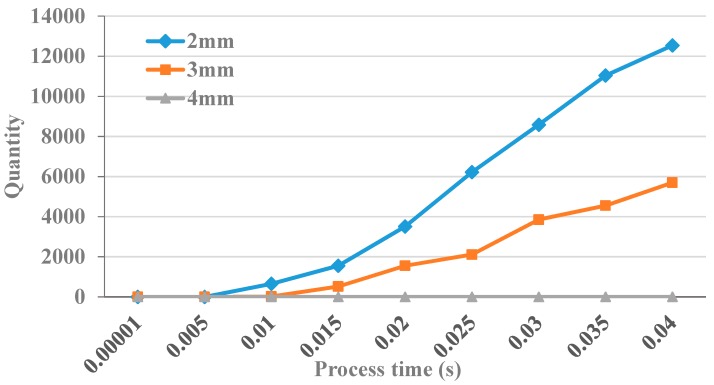
The quantity variation of debris.

**Figure 18:**
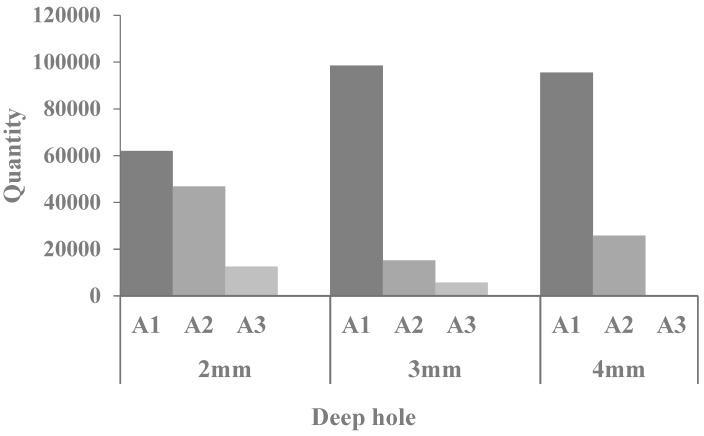
The quantity of debris distribution.

When the amplitude is 5 μm and the frequency is 20 kHz, Figure 17 shows that when the hole depth is 2 mm, 3 mm, and 4 mm, respectively, the number of debris changes with time in A3 region, Figure 18 shows the distribution of debris in A1, A2, and A3 areas at the processing time of 0.04 s.

By analyzing the simulation results, it can be obtained that debris in the machining gap are more and more difficult to remove with the increase of hole depth. As can be seen from Figure 17 and Figure 18, when the depth of the hole is 2 mm, the debris begin to move into A3 area very quickly under the impact of flushing liquid and ultrasonic vibration, It can be seen that at 0.04 s, the number of debris in A3 area is 12,542, and the removal rate is about 10%. When the hole depth is 3 mm, at 0.04 s, the number of debris in A3 area is 2300, and the removal rate is about 1.3%. When the hole depth increased to 4 mm, there was little the debris in A3 area. This is because the height of debris in the side clearance is affected by ultrasonic vibration, and only under the impact of ultrasonic vibration for a certain period of time can it rise to the effective area of flushing liquid, and then discharged to the A3 area. The simulation results show that the removal efficiency of debris is improved under the combined impact of ultrasonic vibration with flushing liquid. However, as the hole depth increases, it is necessary to change the parameters, such as flushing speed and ultrasonic vibration amplitude, so as to enable better discharge of debris.

## 5. Conclusions

This paper applies the Fluent software to simulate the process of the distribution and movement of debris in small hole machining with ultrasonic assisted EDM. The effect of different amplitudes and frequencies of vibration on the debris movement, the debris distribution in each region, and the flow velocity distribution in the bottom gap are quantitatively researched. Through the analysis, it is found that tool electrode assisted with ultrasonic vibration promotes inter-electrode working fluid circulation, which is beneficial to the removal of debris in the process. Moreover, as the frequency and amplitude increase, the electrode of disturbance is acute in the bottom gap, the flow rate of working fluid flowing through the machining gap increases, and the debris along with the working fluid are more likely to be removed from the machining gap. In addition, the low ultrasonic vibration machining parameters are weak to accelerate the removal of debris. With the increase of the aspect ratio of the hole, the debris removal becomes difficult. Even with the existence of ultrasonic assisted vibration, the removal of debris is poor when the aspect ratio of deep hole is 4. Increasing the vibration parameters obviously improves the removal of debris in the machining gap, thus ensuring the processing stability and improves the process efficiency.

## Figures and Tables

**Table 1:** Simulation parameters set in Fluent.

Solver	Time	Working Fluid	Velocity of Flushing	Ultrasonic Vibration	Flow Field Model	Time Step	Steps
Pressure	Transient	Deionized Water	2 m/s	Call the DEFINE_CG_MOTION Macro Function.	Laminar-Model	0.00001	4000
